# Distinct frequency patterns of *LILRB3* and *LILRA6* allelic variants in Europeans

**DOI:** 10.1007/s00251-022-01286-1

**Published:** 2022-11-30

**Authors:** Arman A. Bashirova, Wojciech Kasprzak, Colm O’hUigin, Mary Carrington

**Affiliations:** 1 Basic Science Program, Frederick National Laboratory for Cancer Research, Frederick, MD USA; 2 Laboratory of Integrative Cancer Immunology, Center for Cancer Research, National Cancer Institute, Bethesda, MD USA; 3 Ragon Institute of MGH, MIT and Harvard, Cambridge, MA USA

**Keywords:** LILR, Polymorphism, Non-allelic homologous recombination, Copy number variation

## Abstract

The leukocyte immunoglobulin–like receptor (*LILR*)*B3* and *LILRA6* genes encode homologous myeloid inhibitory and activating orphan receptors, respectively. Both genes exhibit a strikingly high level of polymorphism at the amino acid level and *LILRA6* (but not *LILRB3*) displays copy number variation (CNV). Although multiple alleles have been reported for both genes, limited data is available on frequencies of these alleles among humans. We have sequenced *LILRB3/A6* exons encoding signal peptides and ectodomains in 91 healthy blood donors of European descent who carry one or two copies of *LILRA6* per diploid genome*.* Analysis of haplotypes among individuals with two *LILRA6* copies, representing the majority in this cohort (*N* = 86), shows that common *LILRB3* and *LILRA6* alleles encode some distinct amino acid sequences in homologous regions of the receptors, which could potentially impact their respective functions differentially. Comparison of sequences in individuals with one vs. two copies of *LILRA6* supports non-allelic homologous recombination between *LILRB3* and *LILRA6* as a mechanism for generating *LILRA6* CNV and *LILRB3* diversity. These data characterize *LILRB3/LILRA6* genetic variation in more detail than previously described and underscore the need to determine their ligands.

**Supplementary Information:**

The online version contains supplementary material available at 10.1007/s00251-022-01286-1.

Leukocyte immunoglobulin–like receptors (LILRs; also known as ILTs and LIRs) comprise a family of inhibitory and activating receptors encoded by a gene cluster within the leukocyte receptor complex on human chromosome 19q13 (Anderson and Allen 2009; Wilson et al. 2000). Among 11 family members, inhibitory LILRB3 (ILT5, LIR3) and activating LILRA6 (ILT8) represent a unique homologous receptor pair characterized by highly polymorphic ectodomains (Bashirova et al. 2014; Borges et al. 1997; Colonna et al. 1997; Lopez-Alvarez et al. 2014; Torkar et al. 2000). LILRB3 was reported to be expressed primarily by myeloid cells based on flow cytometry (Colonna et al. 1999; Sloane et al. 2004; Tedla et al. 2003; Yeboah et al. 2020). Although cell surface antibody staining does not allow distinction between LILRB3 and LILRA6, it appears that LILRB3 expression dominates since antibody ligation results in inhibition of myeloid cells (Sloane et al. 2004; Yeboah et al. 2020) and mass spectrometry analysis of neutrophil lysates did not detect LILRA6, but did detect LILRB3 (Zhao et al. 2020).

Natural ligands for LILRB3 and LILRA6 are uncertain. Although recent work suggested that LILRB3 interacts with HLA class I (Ayukawa et al. 2021), this does not agree with structural predictions (Willcox et al. 2003) and other studies that failed to detect this interaction (Allen et al. 2001; Colonna et al. 1998; Jones et al. 2011). Other reported LILRB3 binding partners include angiopoietin-like proteins (Zheng et al. 2012), *Staphylococcus aureus* (Nakayama et al. 2007), and a cytokeratine-8-associated ligand on necrotic cells (Jones et al. 2016). Given recent work suggesting LILRB3 as a potential therapeutic target (Wu et al. 2021; Yeboah et al. 2020), it is important to further understand the biology of LILRB3 and LILRA6 receptors, including regulation of their function.

Genetic polymorphism may determine in part LILRB3 and LILRA6 ligand engagement. Genes encoding these receptors generate multiple protein variants differing in their ectodomains (Bashirova et al. 2014; Colonna et al. 1997; Hirayasu et al. 2021; Lopez-Alvarez et al. 2014). In addition, *LILRA6* exhibits copy number variation (CNV), which appears to be the result of non-allelic homologous recombination (NAHR) (Bashirova et al. 2014; Lopez-Alvarez et al. 2014) and may regulate the balance between inhibitory and activating signaling. Due to the homology between *LILRB3* and *LILRA6*, as well as *LILRA6* CNV, genotyping SNPs in these genes is challenging. Published allelic variants were derived from studies of ≤ 20 individuals and publicly available genome-wide data are not reliable for SNP analysis in these genes. To estimate frequencies of alleles at the *LILRB3* and *LILRA6* loci, we sequenced the exons of *LILRB3* and *LILRA6* encoding signal peptides and ectodomains in 91 individuals of European descent with one or two copies of *LILRA6* per diploid genome from a cohort of healthy blood donors described in our previous work (Bashirova et al. 2014).

*LILRB3* and *LILRA6* genomic fragments encompassing the first seven exons were amplified using gene-specific primers and each exon was sequenced by Sanger sequencing (Supplementary Table). Detailed information regarding *LILRB3* and *LILRA6* variation at the nucleotide and protein level is found in the Supplementary file. Among individuals with two *LILRA6* copies (*N* = 86), a total of 53 distinct SNPs were detected in each gene that resulted in amino acid changes at 32 and 30 positions for LILRB3 and LILRA6, respectively. Nonsynonymous SNPs with frequencies > 5% in either *LILRB3* or *LILRA6* are listed in Fig. 1A (*N* = 39). The SNPs involved in amino acid changes at identical positions in the homologous regions of the two receptors showed substantially different frequencies in some cases (Supplementary Fig). These include several positions across the signal peptide and D1-D4 domains (shown in red in Fig. 1A, B). This distinct polymorphism in the signal peptide and ectodomain may potentially result in differential intracellular transport and/or ligand binding capacity of LILRB3 and LILRA6.

**Fig. 1:**
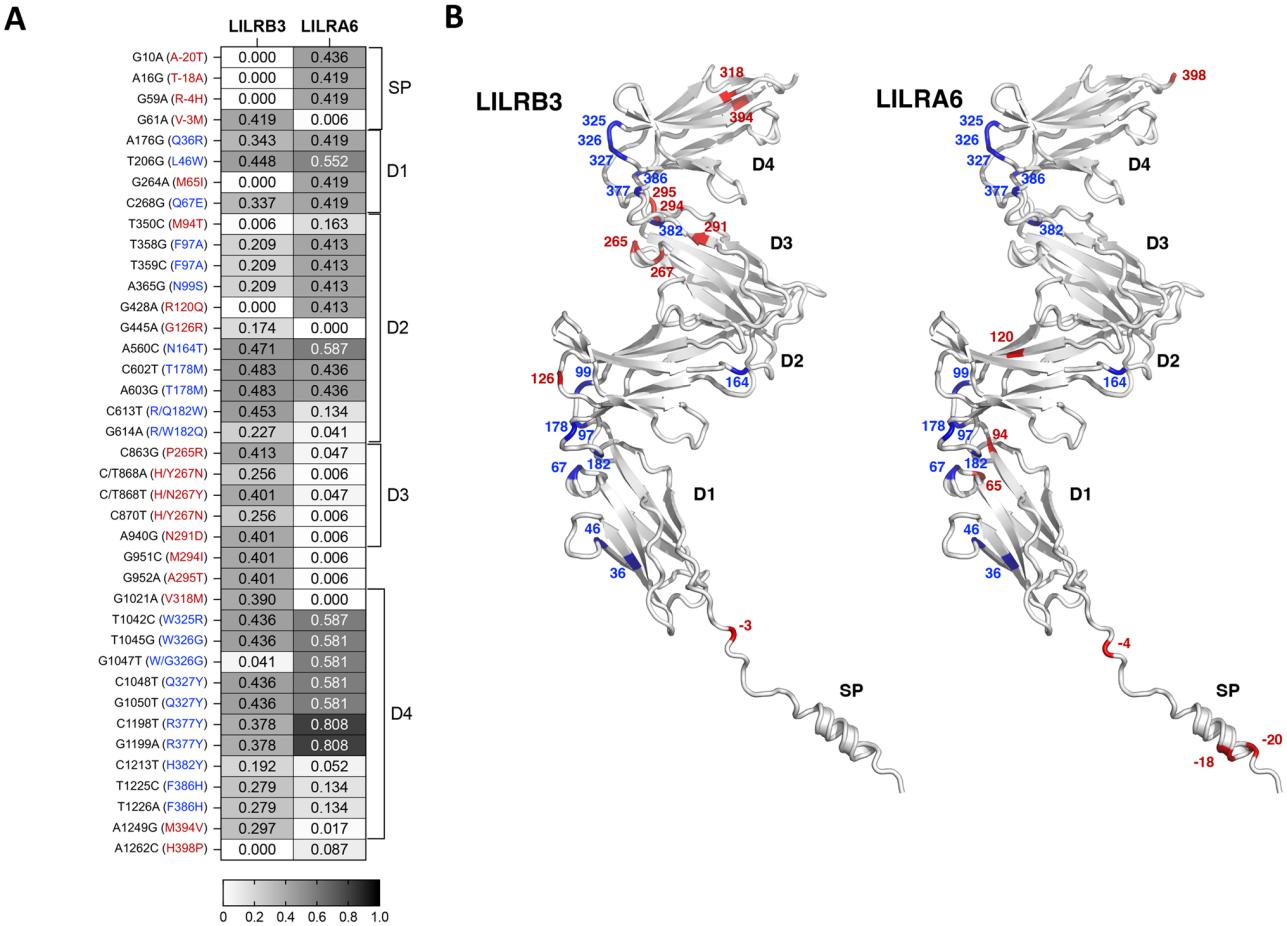
Polymorphism in LILRB3 and LILRA6 observed in individuals with two copies of *LILRA6*. (**A**) The heatmap shows nonsynonymous SNPs in exons encoding the signal peptide (SP) and ectodomain, including Ig-like domains D1-D4. Only SNPs with frequencies > 5% in at least one of the genes are included. The nucleotide variants and corresponding amino acids (in parentheses) are shown on the left. Protein domains are depicted on the right. Frequencies provided represent the second variant (e.g., the frequency of A is shown for G10A). Nucleotide positions are relative to the ATG start codon and amino acid positions correspond to the mature protein. Amino acid positions that have variants with > 5% frequency in both genes are highlighted in blue and those that have variants with > 5% only in one gene are shown in red. (**B**) Ribbon diagrams of signal peptides and ectodomains in LILRB3 and LILRA6 depicting polymorphic sites highlighted in blue and red as defined in (**A**). The 3D structures correspond to 421 amino acid–long fragments of the AlphaFold models O75022 for human LILRB3 (https://alphafold.ebi.ac.uk/entry/O75022). Rendering was performed with a custom script in PyMOL 2.4 software package by Schrodinger (https://pymol.org/2/)

To estimate the frequency of protein variants (allotypes) in individuals with two copies of *LILRA6*, we inferred haplotypes composed of nonsynonymous SNPs using Haploview software (Barrett et al. 2005). Figure 2 shows six LILRB3 and four LILRA6 allotypes that were estimated at frequencies > 3% in our dataset. All of these were previously described in at least one study that determined alleles by cloning cDNA in small groups of individuals (Bashirova et al. 2014; Hirayasu et al. 2021; Lopez-Alvarez et al. 2014). Despite the homology between LILRB3 and LILRA6, none of the protein allotypes (i.e., signal peptide/ectodomain region) were identical between the two receptors. Phylogenetic analysis of amino acid sequences did not separate LILRB3 and LILRA6 variants into distinct clades, although the most common variants, LILRB3_1 and LILRA6_1, were the most divergent (Fig. 3). Overall, the phylogenetic tree is consistent with dynamic exchange of the genetic material between the two genes as a result of NAHR. The maximal divergence found in pairwise comparisons of protein sequences for alleles within each locus is 4.7% (LILRB3_1 and LILRB3_2) and 4.2% (LILRA6_1 and LILRA6_4). In contrast, divergence between the LILRB3 and LILRA6 allotypes can be considerably less, ranging from 1.6 to 5.4%. A homologous gorilla LILRB3 sequence (XP_030859787) averages 8.7% divergence from the human sequences (both LILRB3 and LILRA6). The human and gorilla lineages are thought to have separated some 8 million years ago. Thus, the substitution rate for these *LILR* genes approximates 1% divergence per million years. On this basis, the 4.7% divergence found among LILRB3 allotypes represents several million years divergence between allelic sequences.

**Fig. 2:**
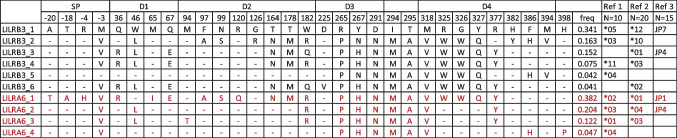
LILRB3 and LILRA6 allotypes in individuals with two copies of *LILRA6*. Phase of the nonsynonymous amino acid variants was estimated using Haploview (first 436 amino acids). Only haplotypes with frequencies > 3% are included. Dashes indicate identical amino acids to the reference sequence LILRB3_1 encoded by transcript ENST00000445347. Domains corresponding to amino acid positions are shown on top. Matching variants from previous work are shown on the right: ref 1 – Bashirova et al. (2014), ref 2 – Lopez-Alvarez et al. (2014), ref 3 – Hirayasu et al. (2021)

**Fig. 3:**
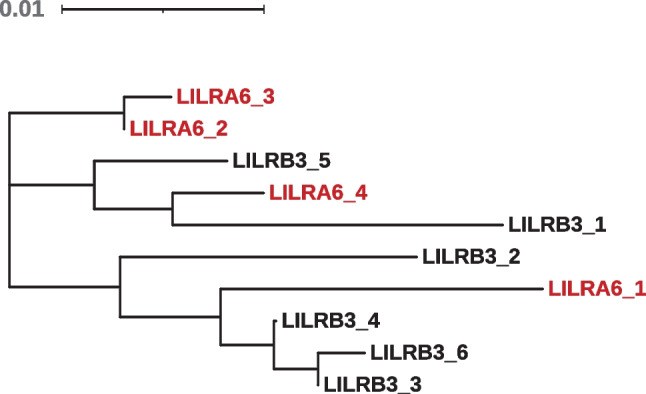
Phylogenetic relationship between LILRB3 and LILRA6 allotypes estimated in individuals with two copies of *LILRA6*. A neighbor-joining tree was constructed based on alignment of homologous fragments of the proteins (first 436 amino acids). Poisson-corrected evolutionary distances between sequences were computed from amino acid identities. The tree is rooted at its midpoint and the scale indicates the proportion of amino acid divergence between sequences

Common LILRA6 allotypes were observed among the five individuals with a single *LILRA6* copy, including two LILRA6_1, two LILRA6_3, and one LILRA6_2 allotypes. Interestingly, LILRB3 sequences in these same donors showed heterozygosity at positions -20 (A/T), -18 (A/T), -4 (H/R), or -94 (M/T) (Fig. 4A), which were either fixed or rarely polymorphic in LILRB3 (i.e., -20A, -18 T, -4R, and 94 M) among people with two copies of *LILRA6* (Fig. 1A). Comparison of the LILRB3 sequences in these five individuals with the inferred LILRB3 and LILRA6 allotypes shown in Fig. 2 suggested the presence of one recombinant LILRB3 allotype in each person that is partially identical to LILRA6_1 or LILRA6_3 (see Fig. 4A, B as an example). The recombinant *LILRB3* gene in each of these five subjects is very likely to have been generated by an ancestral NAHR event between a *LILRA6* gene and a *LILRB3* gene on homologous chromosomes, which eliminated the *LILRA6* gene in the process (Bashirova et al. 2014; Lopez-Alvarez et al. 2014). Thus, we propose that in each of these five individuals, the recombinant *LILRB3* gene (encoding the signal peptide and at least part of the ectodomain derived from LILRA6 and the remainder (including the signaling domain) derived from LILRB3) is present on the haplotype that is missing the *LILRA6* gene. These data strongly support NAHR as a mechanism for *LILRA6* CNV and *LILRB3* diversification.

**Fig. 4:**
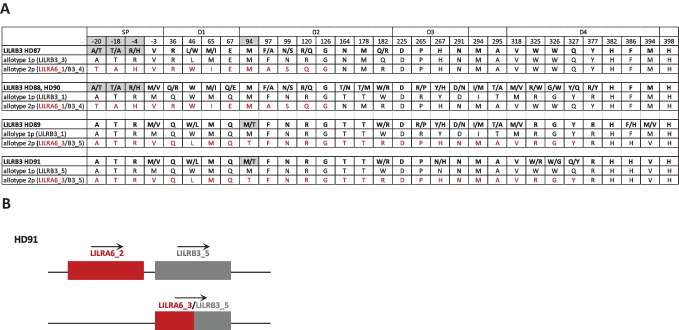
LILRB3 amino acid sequence and predicted allotypes in individuals with one copy of *LILRA6*. (**A**) LILRB3 amino acid sequences encoded by genotypes of five individuals (HD87–HD91) are shown in bold. Gray highlights indicate positions that are fixed or rarely polymorphic in LILRB3 among 86 individuals who have two *LILRA6* copies (i.e., one per chromosome). Predicted allotypes (allotype 1p and allotype 2p) encoded by the two *LILRB3* alleles in each individual were determined by comparing the amino acid variants present in each of the five donors with common allotypes shown in Fig. 2. This comparison suggests the presence of a recombinant *LILRB3* gene product (allotype 2p) containing LILRA6_1-like sequences in HD87, HD88, and HD90 and LILRA6_3-like sequences in HD89 and HD91 in the respective signal peptide/ectodomains. (**B**) Schematic representation of the predicted *LILRB3/LILRA6* genotype in donor HD91. Genes are shown as boxes. Labels above the boxes depict the corresponding protein variants. The top chromosome has a “normal” configuration of the locus containing both *LILRB3* and *LILRA6* that has not undergone NAHR. The homologous chromosome on the bottom is theoretically derived from an ancestral NAHR, as it is missing the *LILRA6* gene and carries a recombinant *LILRB3* gene encoding a molecule identical to LILRA6_3 in its N-terminal fragment (the SP and most of the ectodomain) and LILRB3_5 in its C-terminal fragment (remainder of the ectodomain and the transmembrane/cytoplasmic domains), the latter of which defines this receptor as inhibitory LILRB3

Our cohort is limited to individuals of European descent, and it is possible that these genes display population-specific diversity. Recent work indicated the frequent occurrence of a specific group of *LILRB3* alleles, called lineage L3, among 15 Japanese individuals (Hirayasu et al. 2021), whereas this lineage was rarely seen in our cohort (Supplementary file; characteristic variant is 30Q). This population-specific distinction is consistent with the frequencies of rs76998994, a lineage L3-tagging SNP, in gnomAD genomes, which show an allelic frequency of 18% in East Asians vs. 1–2% in European individuals. Also, our dataset herein was somewhat biased by primarily including individuals with 2 copies of *LILRA6*. Previous work has shown that this configuration is present in only 2/3 of European individuals, whereas the other 1/3 have zero or more than 2 copies of *LILRA6* per diploid genome (Bashirova et al. 2014). Given that NAHR, which likely caused the *LILRA6* CNV, results in exchange of genetic fragments between *LILRA6* and *LILRB3*, we expect that individuals with duplications and deletions of *LILRA6* will carry more diverse sequences than that seen in the current study. Thus, more work needs to be done for accurate allelic characterization of this locus.

Our study is the first to estimate allelic frequencies of *LILRB3* and *LILRA6* genes in a sizable population group. Although our dataset may potentially miss some alleles if PCR/sequencing primers contain polymorphic sites affecting DNA amplification, such allelic dropouts would be rare given the consistency of our data with Hardy–Weinberg equilibrium (see Supplementary file) and previously published data (see Fig. 2). Extensive variation at the *LILRB3/LILRA6* locus may translate to inter-individual variation in immune responses and risk of disease, emphasizing the need to determine ligands for LILRB3 and LILRA6 and the strength of their interactions as a function of genetic polymorphism.

## Supplementary Information

Below is the link to the electronic supplementary material.

Supplementary file1 (DOCX 55 KB)

Supplementary file2 (XLSX 79 KB)
