# Supplementary material for: Distinct frequency patterns of LILRB3 and LILRA6 allelic variants in Europeans
Source: Immunogenetics. 2022 Nov 30;75(3):263–7. doi: 10.1007/s00251-022-01286-1 (PMC10205885; doi:10.1007/s00251-022-01286-1)
Supplement: Supplementary file 1 — Supplementary file1 (DOCX 55 KB) [file 251_2022_1286_MOESM1_ESM.docx]

**Supplementary Table. Primers used for PCR and sequencing.**

| **Primer** | **Sequence** | **Direction^1^** | **Location** | **Procedure** | **Gene** |
| --- | --- | --- | --- | --- | --- |
| ILT_1 | CTTCCTGTGGGCTGAGGATG | forward | 5' upstream of exon 1 | PCR^2^ | LILRB3 LILRA6 |
| ILT_15 | CCAGATGTCCTGTGTTTGCTG | reverse | exon 8 | PCR^2^ | LILRB3 |
| ILT_26 | TGAGGCTCCACCACGCTGA | reverse | exon 8 | PCR^2^ | LILRA6 |
| ILT_109 | TGAGGATGAGACAACC | forward | 5' upstream of exon 1 | sequencing exon 1 | LILRB3 LILRA6 |
| ILT_64 | GGACCTGCTCAGGCTT | forward | intron 1 | sequencing exon 2 | LILRB3 LILRA6 |
| ILT_66 | AGCTGGGATCTGAGCG | forward | intron 2 | sequencing exon 3 | LILRB3 LILRA6 |
| ILT_5 | CCGCTGCCACTATTACAGCT | forward | exon 3 | sequencing exon 4 | LILRB3 LILRA6 |
| ILT_115 | GGAGATTCTGCCCTCAG | forward | exon 4 | sequencing exon 5 | LILRB3 LILRA6 |
| ILT_68 | GAGAGACAGTGAGACCTG | forward | intron 4 | sequencing exon 5 | LILRB3 LILRA6 |
| ILT_71 | AGGTGTCAGCTCAGAGC | forward | intron 5 | sequencing exon 6 | LILRB3 LILRA6 |
| ILT_116 | TCTCTGAGCTCAAAGG | forward | intron 6 | sequencing exon 7 | LILRB3 LILRA6 |

^1^Direction relative to transcription

^2^PCR was performed with Platinum Taq polymerase (Thermo Fisher) according to manufacturer’s protocol using ~ 50 ng of genomic DNA in 20 μL total volume with 64°C annealing temperature and 4-minute extension, 40 cycles.

**Supplementary Figure. Frequency patterns of nonsynonymous SNPs in *LILRB3* and *LILRA6* among individuals with 2 copies of *LILRA6*.** Frequencies of nonsynonymous SNPs at identical positions in homologous regions of *LILRB3* and *LILRA6* are shown. Nucleotide positions are relative to the ATG start codon. Allelic frequencies represent the second variant (e.g., the frequency of A is shown for G10A). Data used to generate the graph is provided in Supplemental File.
